# Injury and Return to Work Among Maritime Workers in British Columbia, Canada

**DOI:** 10.1177/10482911251316325

**Published:** 2025-03-04

**Authors:** Barbara Neis, Robert A. Macpherson, Desai Shan, Contessa Small, Cory Ochs, Lillian Tamburic, Christopher B. McLeod

**Affiliations:** 1 Department of Sociology, Memorial University of Newfoundland, St. John's, Newfoundland, Canada; 2 School of Population and Public Health, 8166 The University of British Columbia, Vancouver, BC, Canada; 3 Faculty of Medicine, 7512 Memorial University of Newfoundland, St. John's, Newfoundland, Canada; 4 7512 Memorial University of Newfoundland, St. John's, Newfoundland, Canada

**Keywords:** occupational health, maritime workers, return to work, British Columbia, Canada

## Abstract

Maritime occupations encompass seafaring, fishing, marine aquaculture, and longshore work. These non-standard occupations tend to be hazardous with high injury rates. They are associated with varying levels of seasonality, shift work, geographic mobility, and different types of remuneration, posing unique challenges when recovering from work-related injury and illness. Occupational health and safety is under-researched in these sectors. Furthermore, little research exists on return to work (RTW) after injury among maritime workers. This paper presents findings from a mixed methods research program designed to provide insight into injury, compensation and RTW experiences among maritime workers in the Canadian province of British Columbia (BC). Research methods include the analysis of provincial workers’ compensation data, data from an anonymous online survey of injured/ill BC maritime workers and from semi-structured interviews with injured workers and key informants. Analysis of workers’ compensation data shows high rates of serious injuries, longer disability duration, and high rates of deemed RTW, particularly in fishing. Survey findings suggest a relatively low percentage of workers file claims for workers’ compensation to WorkSafeBC. Interview data highlight some of the challenges that may explain under-reporting, longer disability duration, and relatively poor RTW outcomes. Policy relevant concerns and areas for future research relevant to understanding and addressing some of the identified RTW challenges associated with these sectors are presented.

## Introduction

In Canada and globally, maritime workers play critical roles in providing access to seafood, transporting essential commodities to domestic and global markets, and in transportation of people between coastal areas as with ferry workers. Workers in these sectors, including commercial fishing, seafaring, marine aquaculture, and longshore work, encounter multiple hazards and have higher than average injury and fatality rates but health and safety research in these sectors is limited. Commercial fishing takes many different forms but is one of the most hazardous occupations globally with high accident and fatality rates, including in countries in Europe and North America.^1–4^ Seafarers work on board diverse types of vessels including container and other types of cargo ships, cruise ships, ferries and tugboats, and engage in work rotations ranging from months to days at a time in national and, for some, international waters. Seafarers also experience high injury and fatality rates. For instance, the fatal accident rate among seafarers in the UK (at 14.5 per 100,000 workers) was 21 times the rate in the general British workforce between 2003 and 2012. In Canada, the fatal accident rate among seafarers was 22 per 100,000 between 1996 and 2005,^5,6^ approximately four times higher than the rate for all workers across Canada (5.9 per 100,000 between 1993 and 2005).^7 p 24^

Occupational health in marine aquaculture is poorly studied globally but, where good data exist, diverse hazards and high injury and fatality rates have been documented.^8–11^ Longshore work in ports, critical infrastructure linking marine transport and land-based supply chains, is also understudied but hazardous. The introduction of containerization over the past 50 years may have reduced the number of injuries in container ports relative to the past, but injury rates remain high among the much smaller remaining workforces.^12,13^ Furthermore, not all ports are container ports as many still handle bulk cargoes of various kinds so continue to expose workers to longstanding hazards associated with this sector.

These sectors play an indispensable role in global supply chains and food production but are often relatively small (in terms of employment), dispersed and somewhat invisible, including in Canada, the United States and Europe. Within maritime work, injuries and fatalities arise from multiple and often interacting factors. For those working at sea, these include marine weather and sea conditions, work on mobile and moving platforms, vessel instability, living and working in confined and noisy spaces, limited access to safe wharves and harbors, issues with safety training, use and appropriateness of personal protective equipment (PPE), demanding work schedules and intermittent rest hours, and remoteness from shore and healthcare facilities.^14–16^ While less remote from shore and healthcare facilities, as indicated in their job description, longshore workers also often work on moving platforms moving diverse types of cargo around on docks, in vessel holds and between ship and shore.^17^

To our knowledge, there is almost no research on return to work (RTW) after injury in these sectors. These characteristics of maritime work may play a role in RTW. For instance, work-related mobility—i.e., extended/complex mobility to work, has been shown to influence both injury risk and RTW after injury among inter and intrajurisdictionally mobile workers in Canada and elsewhere.^18^ Like others who are employed in non-standard work environments located at some distance from their homes and thus are mobile to and often within work, maritime workers may confront not only multiple hazards but also challenges accessing medical care, injury, and illness compensation, and with RTW. Furthermore, many maritime workers are precariously employed as with seasonally employed fishing and aquaculture workers and those with casual employment with no guaranteed hours of work or union protection may be dispatched to transient employers, as with some seafarers and longshore workers. As indicated elsewhere (including in this special issue), these types of precarious employment may lead to higher injury/illness risk,^19^ under-reporting of injuries and illness and compensation claim suppression; they can also constrain access to options and supports for RTW thus delaying or preventing RTW. Claim suppression is defined as:

any overt or subtle action by an employer or its agent that has the purpose of discouraging a worker from reporting a work-related injury or disease or from claiming workers’ compensation benefits to which he or she would likely be entitled. In the absence of inducement or pressure not to report an incident to a workers’ compensation board or not to claim benefits, under-reporting and under-claiming alone do not constitute claim suppression.^20^

In Canada, maritime work takes place primarily on Canada's east and west coasts with some also taking place on the Great Lakes and in the Arctic. Workers’ compensation is almost exclusively a provincial responsibility with some federal responsibility for some maritime workers such as seafarers. This paper draws on findings from a recent mixed methods study with quantitative and qualitative components that explore hazards, injuries and RTW among injured maritime workers in the Canadian province of British Columbia (BC) which encompasses Canada's Pacific coast. Data are derived from (1) an analysis of WorkSafeBC workers’ compensation injury and RTW data for seafarers, longshore workers, fishermen, and aquaculture workers in BC; (2) a 2023 anonymous online survey targeting BC maritime workers in these sectors who had experienced occupational injuries or illnesses related to that work in the past 10 years; and from (3) injured worker and key informant interviews conducted in 2023-2024.

We have selected BC for this study for a number of reasons. Firstly, in BC, maritime work plays an important role in the economy. In 2021, longshore work employed 12,670 workers, seafaring employed 6,260 workers, and fishing and aquaculture employed 2,660 and 1,555 workers, respectively.^21^ Between 2011 and 2021, although employment decreased by 32% in fishing, 36% in aquaculture, and 4.4% in seafaring, it increased by 22.2% in longshore work.^21,22^ Secondly, in BC as opposed to Canada's Atlantic coast, there has been no research on occupational health among maritime workers. Thirdly, unlike the situation for researchers in Atlantic Canada whose background is mainly in fisheries and aquaculture occupational health, and where there is very limited and uneven access to compensation data, researchers involved in this study have unique access to its data on claims and RTW in BC through an agreement with WorkSafeBC and have used its data for multiple studies of RTW in other sectors. Finally, it is worth noting that a new study focusing on RTW in Atlantic Canada is now underway involving some of this study's authors and key elements of the research design used here thus hopefully providing the basis for future comparisons.

In BC, as elsewhere, employment situations are diverse within and across the maritime sectors: seafarers work on ferries, tugboats, cargo and other vessels, and barges; longshore workers are employed in large (Vancouver) and smaller ports spread along the BC coast and in the Fraser Delta and deal with a variety of cargoes and related situations. Employment in fisheries and aquaculture is also diverse and dispersed along the coast. There are unions representing seafaring and longshore workers but not all workers in these sectors are unionized—there are, for example, many casual workers employed in the longshore sector. Most aquaculture workers are not unionized in either BC or other parts of Canada and while some are employed by large, vertically integrated companies on salmon farms, many are employed in small and medium-sized enterprises in the shellfish sector.^23^ In BC, the future of employment on salmon farms is also uncertain due to government plans to ban ocean-based net pen farming. As elsewhere, the fishing sector is characterized by a high rate of self-employment (36.8% of all employed fishing workers 2011-2021). Numbers of marine fishing workers have declined in recent years on both coasts of Canada, but particularly in BC and average incomes for these workers are lower in BC than in Atlantic Canada. Incomes are insecure for most Canadian fishing workers due to seasonality, share-based wages and other factors. This contributes to transience, particularly among crew members in the sector.^24,25^ Employment Insurance (EI) payments are an important source of income among seasonal fishing workers. Rules governing EI eligibility and benefits vary within the sector but for self-employed fishers are not based on a combination of wages and weeks of work, but on income from landings. When coupled with short seasons, the catch share system and the fact that time off on workers’ compensation does not count towards EI eligibility, their situation may contribute to pressure to keep fishing, even after injury.^26^ On Canada's east coast unionization levels among fishing workers are variable (high in Newfoundland and Labrador, low elsewhere). We have been unable to find statistics on unionization levels and trends in BC's fishing industry. Overall, although there is some variation, many workers in BC's maritime sectors appear to experience some degree of employment precariousness and potentially other situations of vulnerability such as working remotely and in transient, mobile work sites.

## Methods

WorkSafeBC has a prevention and rehabilitation mandate and administers claims for workers who are adjudicated to have been injured at work. During the study period, between 94% and 98% of BC's provincial workforce was covered under this no-fault compensation system, funded primarily by insurance premiums paid by registered employers.^27^ In addition to compulsory coverage, workers who are self-employed proprietors or partners in a partnership can apply for Personal Optional Protection (POP) coverage.^28^ For the workers’ compensation claims analysis, administrative data from WorkSafeBC were extracted and analyzed by co-authors Macpherson, Tamburic and McLeod. These data were for workers aged 15-64 with accepted claims for injuries and illnesses that occurred between January 1, 2010, and December 31, 2021. This included information on worker sex, age, and claim type (time-loss claim and serious injury claim). Time-loss claims are claims with costs related to at least one of the following benefit types: short-term disability benefits (STD), long-term disability benefits (LTD), or survivor benefits (fatal). Serious injuries are time-loss claims that represent a serious medical diagnosis (one of 455 selected International Classification of Diseases, Ninth Revision [ICD-9 codes]), a potentially-serious diagnosis (one of 398 selected ICD-9 codes) with a longer recovery period of 50+ days paid (10+ weeks off work), and includes work-related death claims.^29^ Data were aggregated into sex (male/female), age group (15-64), industry sector, and year. To calculate injury claim rates per 1,000 workers across maritime sectors, denominator data (employed labor force) were extracted from publicly available tables from Statistics Canada's 2011 National Household Survey,^22^ the 2016 Census of Population,^30^ and the 2021 Census of Population.^21^ Aggregated (group-level) counts of fishing, aquaculture, marine transportation (referred to below as seafarers), and marine transportation support (referred to below as longshore workers) sectors were created for sex, age group, year using WorkSafeBC classification units and corresponding 4-digit North American Industrial Classification System (NAICS) industry codes (a full list of classification units and NAICS industry codes are listed in Supplemental Table 1). Claims for self-insured employers without classification units (e.g., BC Ferries) were also mapped to corresponding NAICS industry codes. To meet requirements of the *Statistics Act*, denominator and numerator cell sizes with less than five were suppressed.

To examine work disability and RTW outcomes of maritime workers, all accepted time-loss claims from the same classification units were extracted from 2010 to 2020. RTW outcomes were examined using categorical and continuous outcomes. The categorical work disability outcome was the claim outcome after 1 year of follow-up and included: RTW; deemed RTW (fit for RTW according to WorkSafeBC but for various reasons have not yet done so); non-RTW and still on claim. The continuous outcome was work disability days, measured as the total number of calendar days from the date of injury to RTW or deemed RTW with no further-time loss or claim reopening within 1 year of injury. Calendar days for workers without a RTW event or still on claim were censored at 365 days. This measure is consistent with previous research using similar data, albeit with a shorter follow-up in order to retain as many claims as possible.^18,31^ As this analysis did not require denominators and associated data limitations, information on injury diagnosis, pre-injury earnings, shift work, and multiple job status were used in the analysis. Lastly, quantile regression models were used to estimate the differences in work disability days for maritime workers at different points in the distribution of disability duration (25^th^, 50^th^, and 75^th^ percentiles). Model estimates were also used to calculate adjusted predictions for each worker, holding other covariates at their mean values.^32^ Data preparation and analyses were conducted using Stata 16.1 (Stata Corp, College Station, TX, USA).

The anonymous, online survey and interview components of this study were designed and carried out by co-authors Neis, Shan, and Small. Invitations to participate in the survey were distributed on our behalf by unions and other contacts and were also posted online via social media (Facebook, LinkedIn) and community groups such as the Seafarers’ Wellbeing Facebook page. The survey consisted of six sections with 93 items in total and took on average 25 minutes to complete. Part I invited participants to identify their maritime sector and job type at the time of their most serious work-related injury or illness, and to describe hazards in the sector, as well as occupational and injury/illness characteristics. Part II focused on compensation claims and sources for their injury or illness. Parts III and IV asked them to provide information on access to healthcare after the injury and during recovery, rehabilitation treatments, experiences with workers’ compensation during RTW, and included questions about support from their union (where relevant) and employers. In Part V, they were asked about injury or illness effects on personal and family life including socioeconomic impacts and RTW outcomes and Part VI consisted of sociodemographic questions for use in describing the sample. The survey was launched on May 26, 2023, and closed on September 15, 2023.

Co-author Ochs evaluated the 1,291 completed surveys for inclusion, in discussion with the rest of the team. She conducted a three-phased data cleaning strategy to identify possible fraudulent surveys including those completed by bots and fraudulent participants, in other words, those who claimed to match the inclusion data but did not (Supplemental Table 2 provides a detailed summary of the online survey exclusion strategy and numbers excluded based on each identified issue). Recruitment information for the study was widely distributed, including on social media, and included reference to an incentive to complete the survey of a chance to win one of three $100 gift cards. In total, 193 surveys were included for analysis. There were only a few responses from the aquaculture sector, so fishing and aquaculture results are combined below.

Anonymous survey respondents were given the opportunity to express an interest in participating in a confidential, virtual semi-structured interview and to provide, separate from the survey, their contact information for follow-up. Email addresses for 225 participants were provided via the Qualtrics survey but many of these were generated by bots. After careful review, 77 email addresses were retained for follow up contact with those participants asked to complete a follow-up eligibility form introduced to help screen out those who did not fit the inclusion criteria but were interested in entering a lottery for one of three $100 gift cards advertised as an incentive to recruit survey participants. Eleven responded and completed this form. Six injured workers in total were considered eligible and consented to an interview. Of these six, five were seafarers at the time of their injury and one was injured while working in aquaculture and then moved to seafaring. Virtual interviews with injured workers were conducted online using the Zoom platform between October and December 2023.

Potential key informants were approached as individuals or through key organizations across all four maritime sectors about their interest in volunteering to participate in a confidential, virtual interview. Interview participants were invited to share the interview information with others they thought might be interested in participating. Ten key informants with backgrounds in multiple sectors (some had worked in more than one sector) and in unions, government, labor relations, and other kinds of organizations, some of whom had also experienced work-related injuries in the relevant sectors, participated in key informant interviews between February and June, 2024 (Table 1).

**Table 1.** List of Interviewees and Sectors.

Maritime workers (MW) and key informants (KI) 2023-2024	
MW01	Seafarer—master mariner
MW02	Seafarer—engineer
MW03	Seafarer—engineer
MW04	Seafarer—ferry worker
MW05	Seafarer-deckhand
MW06	Aquaculture—shellfish
KI01	Longshore management
KI02	Longshore labor
KI03	Fishing labor
KI04	Crew management
KI05	Government
KI06	Seafarer labor representative
KI07	Seafarer labor representative
KI08	Seafarer labor representative
KI09	Government
KI10	Fishing safety

Interviews were jointly conducted by a minimum of two co-authors (Small and Neis or Shan) and were recorded with written consent. Recordings were transcribed and anonymized by an external professional transcription company with a signed confidentiality agreement with Memorial University. Transcript analysis consisted of identifying key themes and insights and extracting segments relevant to those themes. This work was led by Small, in consultation with other team members.

## Results

### Quantitative Claims and Injury Rate Analysis

Combining data from each of the census years (2011, 2016, 2021), there was a total of 2,040 WorkSafeBC claims in longshore work, followed by 1,566 in seafaring, 539 in fishing, and 514 in aquaculture (Table 2). Three of the sectors had higher time-loss claim rates than the rate across all BC sectors, with the highest in seafaring (49.0/1,000 workers), followed by aquaculture (42.2), and longshore work (37.9). The fishing sector had a lower time-loss claims rate than all other maritime sectors and all BC sectors (21.7) but a higher serious injury claims rate than both groups. Increasing age was associated with lower time-loss claim rates, particularly in the aquaculture, seafaring, and longshore sectors.

**Table 2.** Claim Counts and Rates by Sector.

		All claims		Time-loss claims		Serious injury claims	
	Workers	Count	Rate	Count	Rate	Count	Rate
Fishing	14,345	539	37.6	311	21.7	96	6.7
Aquaculture	4,645	514	110.7	196	42.2	22	4.7
Seafaring	19,410	1,566	80.7	952	49.0	106	5.5
Longshore	33,835	2,040	60.3	1,283	37.9	176	5.2
All BC sectors	6,562,610	365,176	55.6	156,435	23.8	18,964	2.9

*Note.* Rate per 1,000 workers.

During the study period for work disability analysis (2010-2021), there was a total of 9,663 time-loss claims, with longshore work accounting for the largest proportion (47.9%), followed by seafaring (33.4%), fishing (10.9%), and aquaculture (752, 7.8%) (Table 3). In fishing, over one-third (34.3%) of claims were in trawl fishing, followed by longline and trap fishing (27.3%). In aquaculture, the majority (59.3%) were from fin fish farming. In seafaring, the majority (58.9%) were for ferry service, followed by barge, tug, or other water transportation of goods. For longshore work, the highest proportion of claims (40.1%) was from marine container terminals. A wide range of occupations were associated with time-loss claims across these sectors (Supplemental Table 3).

**Table 3.** Time-Loss Claim Counts by Sector and Classification Unit.

	N	%
Fishing		
702009—Trawl fishing	360	34.3
702007—Longline and trap fishing	287	27.3
702006—Gillnet and troll fishing	167	15.9
702008—Seine fishing	108	10.3
702005—Dive fishing	70	6.7
702010—Fish packing	59	5.6
Total	1,051	100.0
Aquaculture		
702001—Fin fish farming	446	59.3
702004—Shellfish farming or hand picking	257	34.2
702002—Fish hatchery	49	6.5
Total	752	100.0
Seafaring		
732014—Ferry service	1,901	58.9
732008—Barge, tug, or other water transport of goods (not elsewhere specified)	722	22.4
732024—Log towing	363	11.3
703009—Log booming or marine log salvage	93	2.9
761050—Chartered boat tours	77	2.4
732038—Water taxi or crew transport	71	2.2
Total	3,227	100.0
Longshore		
732026—Marine container terminal	1,856	40.1
732036—Stevedoring	974	21.0
732020—General wharf operations	756	16.3
732025—Bulk terminal	619	13.4
732023—Loading or unloading goods	388	8.4
732040—Harbor commission, port authority, marine piloting	41	0.9
Total	4,634	100.0

Higher proportions of claims were from younger aquaculture workers, compared to the other sectors (Table 4). The fishing sector had a noticeably higher proportion of claims with fractures (17.8%) as well as acute/traumatic injuries, such as burns and lacerations (26.5%) and dislocations, amputations, and crushing injuries (10.7%). In contrast, injuries in the other sectors were characterized by sprain and strain injuries, particularly of the back. A large proportion of injuries in fishing took place during the third quarter of the year potentially reflecting both seasonality and some of the pressures with accessing EI as outlined elsewhere. Preinjury earnings were generally higher in fishing and longshore compared to seafaring and aquaculture. Large proportions of claims were associated with shiftwork (rotating/variable shift), with the exception of aquaculture. Claims in fishing also had a higher proportion of multiple job-holders than the other sectors.

**Table 4.** Claim Characteristics of Time-Loss Claims by Sector.

	Fishing (*n* = 1,051)		Aquaculture (*n* = 752)		Seafaring (*n* = 3,227)		Longshore (*n* = 4,633)		All BC sectors (*n* = 536,892)	
	*N*	%	*N*	%	*N*	%	*N*	%	*N*	%
Sex										
Male	1,003	95.4	612	81.4	2,468	76.5	4,100	88.5	330,366	61.5
Female	48	4.6	140	18.6	759	23.5	533	11.5	206,526	38.5
Age category (years)										
15-24	149	14.2	157	20.9	265	8.2	336	7.3	71,726	13.4
24-34	257	24.5	209	27.8	613	19.0	1,023	22.1	116,668	21.7
35-44	190	18.1	182	24.2	657	20.4	1,189	25.7	116,875	21.8
45-54	276	26.3	136	18.1	977	30.3	1,223	26.4	138,153	25.7
55-64	179	17.0	68	9.0	715	22.2	862	18.6	93,470	17.4
Injury diagnosis										
Fractures	187	17.8	42	5.6	203	6.3	390	8.4	36,428	6.8
Back sprains and strains	132	12.6	218	29.0	868	26.9	1,325	28.6	157,927	29.4
Upper sprains and strains	120	11.4	93	12.4	452	14.0	677	14.6	72,617	13.5
Lower sprains and strains	78	7.4	83	11.0	562	17.4	740	16.0	64,539	12.0
Dislocations, amputations, and crushing injuries	112	10.7	47	6.3	150	4.6	221	4.8	19,048	3.6
Burns and lacerations	279	26.5	153	20.3	572	17.7	801	17.3	115,214	21.5
Concussion	40	3.8	19	2.5	88	2.7	185	4.0	22,341	4.2
MSD and mental health	103	9.8	97	12.9	332	10.3	294	6.3	48,778	9.1
Injury year										
2010	93	8.8	85	11.3	334	10.4	394	8.5	47,001	8.8
2011	89	8.5	74	9.8	353	10.9	403	8.7	48,327	9.0
2012	106	10.1	83	11.0	320	9.9	431	9.3	48,853	9.1
2013	108	10.3	65	8.6	316	9.8	441	9.5	48,074	9.0
2014	95	9.0	73	9.7	290	9.0	435	9.4	47,889	8.9
2015	103	9.8	70	9.3	272	8.4	442	9.5	47,859	8.9
2016	112	10.7	73	9.7	267	8.3	375	8.1	48,956	9.1
2017	84	8.0	47	6.3	266	8.2	408	8.8	50,089	9.3
2018	100	9.5	56	7.4	282	8.7	438	9.5	51,550	9.6
2019	79	7.5	71	9.4	318	9.9	431	9.3	52,672	9.8
2020	82	7.8	55	7.3	209	6.5	435	9.4	45,622	8.5
Injury quarter										
Quarter 1	185	17.6	176	23.4	780	24.2	1,133	24.5	132,201	24.6
Quarter 2	235	22.4	196	26.1	758	23.5	1,133	24.5	130,401	24.3
Quarter 3	427	40.6	197	26.2	887	27.5	1,229	26.5	140,553	26.2
Quarter 4	204	19.4	183	24.3	802	24.9	1,138	24.6	133,737	24.9
Pre-injury income^a^										
Quartile 1	337	32.1	567	75.4	820	25.4	692	14.9	301,152	56.1
Quartile 2	188	17.9	147	19.5	1,302	40.3	779	16.8	143,977	26.8
Quartile 3	222	21.1	31	4.1	867	26.9	1,296	28.0	72,369	13.5
Quartile 4	304	28.9	7	0.9	238	7.4	1,866	40.3	19,394	3.6
Shift work										
Fixed Shift	40	3.8	371	49.3	399	12.4	463	10.0	249,761	46.5
Rotating/Variable Shift	1,011	96.2	381	50.7	2,828	87.6	4,170	90.0	287,131	53.5
Multiple job status										
Single Job	999	95.1	737	98.0	3,145	97.5	4,483	96.8	510,959	95.2
Multiple Jobs	52	4.9	15	2.0	82	2.5	150	3.2	25,933	4.8
Work disability status										
Returned to Work	697	66.3	628	83.5	2,755	85.4	3,979	85.9	457,511	85.2
Deemed RTW	159	15.1	48	6.4	166	5.1	243	5.2	33,409	6.2
Non RTW	47	4.5	14	1.9	61	1.9	96	2.1	11,443	2.1
Still on Claim	148	14.1	62	8.2	245	7.6	315	6.8	34,529	6.4
Continuous variables										
	Median	IQR	Median	IQR	Median	IQR	Median	IQR	Median	IQR
Days to RTW or Deemed RTW	82	33-223	26	10-103	30	10-104	41	13-113	25	7-91
Days to Sustained RTW	117	40-365	27	10-126	33	10-130	46	14-137	28	8-114

*Note.* Columns percentages unless specified. IQR = interquartile range. ^a^Quartiles based on distribution of maritime workers (cut points at $47,586, $69,464, $101,734).

In terms of work disability outcomes, the most evident differences were for fishing compared to the other groups. Notably, only 66.3% of claimants in fishing had experienced RTW at 1 year of follow-up. In contrast, as much as 85% of those in longshore work had experienced RTW (similar to the provincial rate). Much larger proportions of fishing claims were assigned a “deemed RTW” status by WorkSafeBC (15.1%), non-RTW (4.5%) or were still on claim (14.1%) compared to the other subsectors. The combination of RTW and deemed RTW captures workers no longer in receipt of wage-loss benefits. The median number of days (work disability) until a worker reached such a status was highest for fishing (82 days), followed by longshore work (41), seafaring (30), and aquaculture (26). Despite very little differences in the work disability status between longshore workers and the provincial rates overall, the median disability days to RTW/deemed RTW was higher for longshore workers.

Results from the multivariable quantile regression show that, after adjusting for several individual-level characteristics, there were no differences in work disability duration between longshore (the reference group) and aquaculture (Table 5). However, at the 25^th^ percentile, compared to longshore workers, fishing workers had 16.2 additional days of disability and seafarers had 1.8 days less. At the 50^th^ percentile, fishing workers had 37.1 additional days whereas seafaring had 8.3 days less. For longer duration claims (75^th^ percentile), fishing workers had 100 additional days of disability relative to longshore workers, whereas seafaring had 17.1 days less.

**Table 5.** Multivariable Quantile Regression Estimates of Differences in Work Disability Days by Sector.

	25^th^		50^th^		75^th^	
	Coef.	95% CI	Coef.	95% CI	Coef.	95% CI
Difference in work disability days						
Fishing	16.24	(12.79, 19.69)	37.08	(29.44, 44.72)	100	(74.53, 125.47)
Aquaculture	0.58	(−0.65, 1.81)	−1.36	(−5.60, 2.88)	3.89	(−14.78, 22.56)
Seafaring	−1.75	(−2.52, −0.99)	−8.32	(−11.47, −5.17)	−17.11	(−25.82, −8.40)
Longshore	Ref.		Ref.		Ref.	
Predicted work disability days^a^						
Fishing	32.12	(28.72, 35.52)	81.57	(74.16, 88.98)	223.54	(198.52, 248.56)
Aquaculture	16.46	(15.20, 17.72)	43.13	(39.25, 47.01)	127.43	(109.16, 145.70)
Seafaring	14.12	(13.41, 14.83)	36.17	(33.69, 38.65)	106.43	(98.62, 114.24)
Longshore	15.88	(15.09–16.66)	44.49	(41.96, 47.03)	123.54	(116.62, 130.46)

*Note.* Models adjust for sex, age, injury diagnosis, injury year (grouping), pre-injury earnings (quartile), and shift work status.

a Adjusted predictions for each worker, holding the aforementioned covariates at their mean values.

### Online Survey Results

In the online survey sample, the demographic composition differed slightly from that of the claims cohort, with an average age of 36.5 years compared to 42.1 years, and lower proportion of male workers, 74.9% compared to 84.7%, respectively. The sectoral composition of the respondents also differed to the claims cohort, with 41% working as seafarers, 29.4% in fisheries or aquaculture, and 28.4% in the longshore sector at the time of their injury. Only 42.2% resided in BC at the time of their injury or illness with 28.3% residing in Atlantic Canada and 29.5% in some other part of Canada, suggesting a high rate or interjurisdictional mobility in this sample. Among respondents who answered the question on unionization, 66.2% indicated they were non-unionized; 58.6% worked as contract, casual or seasonal workers; and 43.6% as rotational workers. About 89.7% indicating they experienced moderate to extremely severe symptoms in the case of physical injuries and 70.4% indicating the same for occupational illnesses.

Among 181 respondents to the question on compensation claims, 102 people had submitted only one claim for compensation, of which only 38 (37.3%) were submitted to WorkSafeBC as opposed to another agency. An additional 56 respondents submitted claims to more than one agency and of these, 36% or 64.3% included claims to WorkSafeBC. Thus, a total of 74 (46.8% of the 181 respondents) said they had submitted a claim to WorkSafeBC. Of these claims, 57 (77.0%) were successful. This indicates under-reporting to WorkSafeBC in this sample. It may also indicate that data from WorkSafeBC underestimate the injury rate and may also underestimate time-loss if workers are drawing on other sources of compensation for at least part of their time off, although filing a claim does not mean that a claim will be accepted. Other sources of compensation accessed by injured maritime workers included disability insurance provided by employers or unions and federal workers’ compensation, while a small number were compensated by another workers’ compensation board and one through EI.

Most respondents (86.4%) lost time at work due to their work-related injury or illness and according to the online survey, 24.5% of injured workers who took time off work reported being off for six months or more. Of those who selected “yes” for returned to work, only 29% were able to return to full-duty regular work; 25.4% were able to RTW gradually and eventually able to return to full duties. Others reported returning to permanently modified/alternative duties, different work with the same employer, or work with a different employer. RTW challenges included accessing medical treatment, long wait times and commuting distances to access medical care and rehabilitation, and unwillingness on the part of some employers to offer modified work.

### Interview Results

#### “Just Tough It Out”: Claim Suppression and Underreporting

Injured worker and key informant interview transcripts provide important insights into injury experiences, decision-making around compensation claims and RTW challenges. In the case of injuries, they point to under-reporting and the presence of a “just tough it out” culture in these sectors. For instance, an injured maritime worker explained: “So for all the cuts, and stuff like that … I kind of stopped in the clinic and got a stitch or two or whatever, and just kind of carried on. … So, no claim” (MW06, 2023). Another worker explains: “There was probably times when I should have had a lost time injury, just by the nature of that long a career in the marine industry, twisted ankle, yes, toughed it out at sea. What are you going to do? You’re at work” (MW03, 2023).

Seasonal workers, like fishermen, may be particularly unlikely to file for compensation for injuries unless they are too serious to continue working. A key reason for this is likely the shortness of the fishing seasons and the related heavy reliance on EI benefits for their annual incomes. Time off on compensation does not count towards EI eligibility and can thus threaten both eligibility for EI and EI levels since, for fishermen, EI payments are linked to overall income from fish landings. A labor representative key informant explains: “But with fishermen … they don’t report as having injuries where they have to miss the fishing season” (KI03, 2024). Fishermen may also file for compensation after the season is finished, explaining the seasonal variation in WorkSafeBC claims in the sector shown previously. This practice, and the seasonality and dwindling employment options in fishing, may also help account for the higher number of work disability days identified in the compensation claims data for fishing relative to the other maritime sectors, as well as the higher rate of deemed RTW.

Claim suppression may also contribute to under-reporting and failure to file compensation claims, particularly among the precariously employed, who are at greater risk of job loss. As noted by an injured seafarer:

Finally, I had to go to the [Senior Master] and I said, ‘I’ve got a problem with my knee. And I don’t know what to do. … I believe it's from working on this ship and going up and down the stairs.’ And what he told me was he said, ‘You should not report that because you may be laid off.’ I hadn’t put my six months probation in. I didn’t make a WorkSafe claim. That knee problem got worse. I had to take time off because I had to have a knee replacement. … I ended up being off … about five months. But that was all at my own cost. It actually should have been a WorkSafe claim, but it wasn’t. It is what it is. (MW01, 2023)

#### Difficulties and Delays in Accessing Medical Care

Injured workers and some key informants reported difficulties and delays in accessing medical care including after the injury and during recovery. For those injured offshore, particularly in offshore and remote coastal areas there could be significant challenges getting them back to port. A seafarer labor representative (key informant) said:

Working on the ferries, other than up North here at [company], if there's an incident on the ship, be it an injury to a crew member or a passenger, the ship will turn around and get back to dock. And they’ll do that at full speed, and they’ll have an ambulance waiting or whatever. Up North, it's a little bit more challenging, particularly because of the distance away from a port of refuge. Also, within Canada, we don’t have as a robust of search and rescue facilities as the Americans do. On the West Coast we’ve got one search and rescue base and that's out of Comox. And our crews aren’t trained generally to do medevacs off ships. (KI07, 2024)

Even those injured when onshore could experience challenges and delays getting from the dock to their vehicle (which might be in a different community if they work on a vessel), and then to a clinic or emergency room and back to their home.

Not having a family doctor and unwillingness on the part of some doctors to handle compensation cases contributed to problems accessing proper care, as an injured seafarer explains: “… not having a family doctor, also, I feel has played a huge role in the fact that I didn’t get the proper care” (MW05, 2023). He comments further: “… that's one of the first things they ask you when you go to the doctor, ‘Is this WCB related?’ From that moment right there, it's like they don’t want to touch you” (MW05, 2023). Challenges accessing care post injury can be exacerbated by long work rotations and long commutes between home, work and healthcare providers. An injured seafarer explained the difficulty getting access to a clinic while working in a remote area on a rotation schedule of 2 weeks on and 2 weeks off:

So, I work up north, I do the [place to place] run. So, I do two weeks on, and two weeks off. So, working there it's kind of hard. If you get injured or hurt, it's kind of hard to get to say a hospital or a doctor. They do have an OFA [Occupational First Aid] onboard, but it's tough when you’re out, because it's an 18-hour transition between say [port X and port Y]. You may stop in a small port, [lists examples]. And medical care is really hard to get to when you’re up there. (MW02, 2023)

An injured seafarer discussed the challenge of commuting from his home to the place of his physiotherapy treatments with a back injury: “I just don’t think it's something I should be doing—travelling back and forth 35 min each way to go to this physio, five days a week. That's not something you should make somebody with a back injury do” (MW05, 2023). Another explained some of the complications involved in working in remote areas while seeking urban-based medical care in his place of residence:

You need to remember what my situation is here. I live in [place], which is a large population center in the interior of the province, where I was working are remote areas. There is no access, essentially, other than emergency access to medical assistance when I’m on the job. And that's true even today where I work for [company], you got to take two ferries to get to a hospital. And you’ll blow a whole day getting there and back. So essentially, it meant that all of my medical care had to be [place] based, around my residence. (MW01, 2023)

#### Challenges in the Claim and RTW Process

Among those who applied for workers’ compensation, some injured workers reported they felt bullied and mistrusted by players in the system and by employers:

They literally felt like an insurance company, like that's how you are treated, you’re scamming them and they’re trying to find a way to catch you or send you back to work. That was my experience. It just felt really almost bullied in a way … You’re always a liar in their eyes … that's how I felt throughout the whole process. (MW05, 2023)

Some also indicated that compensation payments and company insurance plans did not include compensation for bonuses or overtime which for some were a crucial and substantial part of their income. This could result in economic hardship: “… if I just got my base rate, I would be homeless …. So that's the problem, overtime usually, you think, ‘Oh it's a bonus or whatever.’ To me it's not a bonus, to me it's become my salary” (MW04, 2023). A seafarer adds: “… when I work up North, we get a 25% differential pay. So, the company took that away from me …” (MW02, 2023).

Two seafarers reported difficulty accessing gradual RTW or modified work. A casually employed injured seafarer said: “Oh, first off, I got told, ‘We don’t do [gradual] return to work on the north coast’” (MW01, 2023). A regular full-time employee indicated that, after he filed a claim for his injury, “… they [Human Resources] said, ‘We cannot deal with these, we cannot accommodate these light duties’” (MW04, 2023).

In the case of longshore workers, a key issue with RTW identified by a key informant in longshore worker management, KI01, is the high percentage of casual workers and the dispatch system which sends workers out to shifting port employers depending on ship arrivals. In this system, workers with the least experience and training are sent to the physically most demanding jobs where there are fewer options for modified work. Furthermore, the employer they are working for at the time of the injury is responsible for claims and RTW but may have limited knowledge of and commitment to the worker. This combination of factors, they argued, works against RTW.

So, the very nature of the work, the way it's structured, the way I’ve already described it, is a barrier to return to work. So, I may have worked at one terminal for 1 day and I get injured there, and they’re very reluctant to bring me back on some kind of RTW process because I was only there for 1 day. So, we run into that all the time. And employers have gotten more sophisticated on their RTW processes, but there's still a barrier there for—so see, if you’re a junior, new longshore, chances of getting back in any kind of RTW is pretty minimal (KI01, 2023).

## Discussion and Policy Relevance

Injury and illness rates and RTW are understudied in maritime sectors, even though they are recognized as essential workers sustaining global supply chains and food production. The mobility and precarious nature of their employment, make it difficult for researchers and policy makers to examine and understand the challenges maritime workers have experienced during the workers’ compensation and return to work process. The Maritime Labour Convention, 2006, aims to ensure seafarers’ rights following workplace injuries internationally, but the Convention does not impose obligations on ship owners to ensure injured/sick seafarers’ return to work. For fishing, aquaculture, and longshore workers, their rights and entitlements following workplace injuries are usually subject to their domestic jurisdiction, due to the lack of international labor standards. As documented in current literature, higher injury/illness risk^19^ and under-reporting of injuries and illness and compensation claim suppression are common challenges for international maritime workers.^33–35^ National RTW survey data administered in Australia, for example, has shown how, in comparison to workers covered by state and territorial jurisdictional workers’ compensation systems, those covered under the system specifically for seafarers and maritime workers (Seacare) are less likely to experience rapid RTW (RTW within less than 30 days), even after adjusting for various individual-level factors (sex, age, injury type, self-rated health).^36^

One reason for the neglect of maritime workers’ RTW may be that, in many jurisdictions, maritime employers are not obligated to provide RTW supports for their workers, particularly when the workers are precariously employed. In Australia, however, there is a national scheme of OHS, rehabilitation, and workers’ compensation arrangements that apply to defined seafaring employees called Seacare.^37^ Although it excludes a long list of domestic vessels, Seacare-covered seafarers can return to a shore job or job at sea in a supernumerary capacity within which they may not be required or asked to perform the full duties of a seafarer.^38^

This paper uses findings from a combination of workers’ compensation data from WorkSafeBC and labor force estimates from the Canadian Census of Population and National Household Survey to characterize work-related injury and RTW outcomes among injured BC maritime workers between 2011 and 2021. Data from a 2023 online anonymous Qualtrics™ survey targeting BC maritime workers who had experienced occupational illnesses or injuries in the past 10 years provide insights into injury types, severity, compensation options and issues with access to medical care and RTW challenges. Findings from qualitative interviews with injured maritime workers recruited through the online survey and with key informants provide further insight into issues related to injury response, access to medical care, compensation claiming histories and related RTW challenges.

The variable but relatively high rates of injury, long duration of disability and high rates of deemed RTW in the WorkSafeBC compensation claims data relative to overall provincial rates, signal the hazardous nature of maritime work and provide some indication of significant RTW challenges, particularly among those in fishing. Data from the survey and interviews indicate a relatively low percentage of workers file claims with WorkSafeBC. These data also provide insights into some of the challenges that may explain some of the under-reporting to WorkSafeBC, longer disability duration, and relatively poor RTW outcomes. Those challenges include delayed access to medical treatment for those injured offshore and during recovery; some evidence of claim suppression for the precariously employed, and the relatively low proportion of workers able to return to their pre-injury jobs with the same company in the survey sample. Reduced incomes on compensation and company insurance plans may influence compensation claims, RTW and the extent to which wage replacement meets the needs of maritime workers accustomed to overtime pay and bonuses for certain kinds of work. In the case of fishing, seasonality, declining, and precarious employment and the Canadian EI system for fishing workers may help to explain patterns in the compensation claims data.

Based on the WorkSafeBC claims analysis, it is evident that, despite having lower time-loss claim rates than other sectors (and below the provincial rate overall), when workers in the fishing sector have an accepted claim, it is likely to be for more severe injuries than the other sectors. This was tentatively supported by the qualitative interviews (although few interviewees were in the fishing sector). It is also consistent with other studies that have described how the types of injuries experienced among fishing sector workers are more likely to be fatal or severe.^1^ The severity of injuries among fishing workers may be a factor driving longer work disability duration than other sectors. However, as shown in the regression models, even after adjusting for claim characteristics known to contribute to long work disability (e.g., sex, age, injury diagnosis, earnings, shift work), there was still significantly longer work disability duration among fishing workers. This suggests that other factors may be contributing to differences in RTW for these workers including some flagged in interview findings such as the seasonal nature of their work and the fact that many employed in the fishing industry are working in very small businesses, including self-employed and owner-operators, which face challenges in health and safety management and RTW.^39,40^

Study strengths include the mixed methods design using quantitative and qualitative approaches and ability to triangulate. There are also limitations including, compensation claims data do not completely capture injury rates and what happened with workers not on compensation and not back to work; self selection in survey and interview participants; challenges with bot and inauthentic responses to the online survey; and the small number of interviews, particularly with injured workers, and poor participation by those from the aquaculture and fishing sectors. Unfortunately, the denominator data used for the claim rate analyses was based on employed labor force estimates (whether a person who is in the labor force was employed during the reference week of the survey/census) as opposed to preferred estimates of hours worked, full-time equivalent employees or person-years—these data were not publicly available. As a result, the analysis conducted is likely to have underreported the true injury rate among the workers studied and differences between them.

The study findings have several policy implications.

1. They point to the need for more systematic and ongoing surveillance and investment in injury prevention, access to medical care and to RTW programming in these relatively small, diverse and dispersed maritime sectors.
2. As has been noted elsewhere, lack of clarity around regulatory jurisdiction related to OHS responsibility is an important challenge in effective safety management in maritime sectors with federal and provincial governments having potentially overlapping responsibilities for vessels and ports.^41,42^ WorkSafeBC and Transport Canada (federal government) share OHS jurisdiction over organizations conducting maritime operations. WorkSafeBC has OHS jurisdiction over the business of fishing and vessels, such as ferries, tugboats, boom boats, and vessels used to transport workers that travel between ports in BC. Wharves, docks, and mooring floats are within the OHS jurisdiction of WorkSafeBC, whereas the federal government has OHS jurisdiction over ports and harbors that service interprovincial or international routes.^43,44^ In addition to these potentially gray areas of OHS responsibility, who is responsible for the compensation and RTW of workers once they have a work-related injury is equally complex due to variety of different forms of compensation and insurance available to these workers. Regulatory jurisdictional fuzziness can work against effective surveillance and interventions. B.C.'s Memorandum of Understanding between the federal and provincial governments for coordination of fishing safety could be expanded to include other maritime sectors.^45^
3. Until 2023, B.C. did not have an early and safe RTW (ESRTW) requirement in its legislation. This has now changed but preliminary findings from this study suggest that ESRTW may require tailored interventions to work well in maritime sectors, particularly in fishing.
4. In contrast to the declining fishing sector, the BC longshore sector has grown significantly over the years and not only has higher time-loss injury and serious injury rates than overall rates in BC, but surprisingly showed greater challenges with RTW than the seafaring sector. Given the increasing number of these workers, and their high average salaries,^46^ not only is getting these workers quickly and safely back to work of importance, but so too is preventing their injuries in the first place.
5. Clearer messaging to maritime workers about their rights and the benefits they are entitled to might encourage injury reporting and improve access to compensation.
6. Constraints on access to emergency care, family doctors and rehabilitation services can play an important role in RTW including by contributing to delayed and failed RTW. These challenges are not unique to maritime workers but need to be addressed in efforts to improve RTW.
7. While this requires more research, it appears that safety culture needs to be enhanced in these maritime sectors to reduce injury rates, eliminate underreporting and claim suppression, and identify ways to support RTW.

## Conclusion

RTW in the maritime sector after work-related injury or illness is under-studied globally. This mixed methods program of research is the first empirical study on maritime workers in BC, one of very few in Canada, and is unique in its cross-sector focus and attention not only to injury rates but also RTW. Compensation claims data point to high injury rates and longer periods of disability among those with compensable injuries and illnesses and survey and interview findings provide insights into some of the factors that influence whether workers file for compensation, as well as their access to healthcare and RTW. Those factors are common to delayed RTW among other groups of workers with non-standard employment. While preliminary, findings are consistent with other research on injury rates in these sectors; on the relationship between mobility to and within work and RTW challenges; and with the particular challenges confronting non-unionized and precariously employed workers, including the self-employed and those in small and medium-sized enterprises. Findings support both the need for ongoing surveillance and for sector-specific interventions in order to reduce injury rates and improve RTW options for workers in these economically vital sectors.

## Supplemental Material

sj-docx-1-new-10.1177_10482911251316325 - Supplemental material for Injury and Return to Work Among Maritime Workers in British Columbia, Canada Supplemental material, sj-docx-1-new-10.1177_10482911251316325 for Injury and Return to Work Among Maritime Workers in British Columbia, Canada by Barbara Neis, Robert A. Macpherson, Desai Shan, Contessa Small, Cory Ochs, Lillian Tamburic and Christopher B. McLeod in NEW SOLUTIONS: A Journal of Environmental and Occupational Health Policy

sj-docx-2-new-10.1177_10482911251316325 - Supplemental material for Injury and Return to Work Among Maritime Workers in British Columbia, Canada Supplemental material, sj-docx-2-new-10.1177_10482911251316325 for Injury and Return to Work Among Maritime Workers in British Columbia, Canada by Barbara Neis, Robert A. Macpherson, Desai Shan, Contessa Small, Cory Ochs, Lillian Tamburic and Christopher B. McLeod in NEW SOLUTIONS: A Journal of Environmental and Occupational Health Policy
