# Supplementary material for: Injury and Return to Work Among Maritime Workers in British Columbia, Canada
Source: New Solut. 2025 Mar 4;35(1):47–59. doi: 10.1177/10482911251316325 (PMC11954171; doi:10.1177/10482911251316325)
Supplement: sj-docx-1-new-10.1177_10482911251316325 - Supplemental material for Injury and Return to Work Among Maritime Workers in British Columbia, Canada [file sj-docx-1-new-10.1177_10482911251316325.docx]

**Supplementary Table 1.** Study Sectors and Mapping of Respective North American Industrial Classification System (NAICS) Industry Codes (Denominators) and Classification Units (Numerators) for Claim Rate Analysis.

| **Sector** | **NAICS Industry Code** | **WorkSafeBC Classification Unit** |
| --- | --- | --- |
| Fishing | 1141 - Fishing | 702003 - Commercial Fishing (retired CU) |
|  |  | 702005 - Dive fishing |
|  |  | 702006 - Gillnet and troll fishing |
|  |  | 702007 - Longline and trap fishing |
|  |  | 702008 - Seine fishing |
|  |  | 702009 - Trawl fishing |
|  | 3117 - Fish packing | 702010 - Fish packing |
| Aquaculture | 1125 – Aquaculture | 702001 - Fin fish farming |
|  |  | 702002 - Fish hatchery |
|  |  | 702004 - Shellfish farming or hand picking |
| Seafaring | 4831 - Deep sea, coastal water transportation | 732008 - Barge, tug, or other water transport of goods (not elsewhere specified) |
|  |  | 703009 - Log booming |
|  | 4832 - Inland water transportation | 732014 - Ferry service |
|  | 4872 - Scenic and sightseeing transportation, water | 732024 - Log towing |
|  |  | 732027 - Marine Piloting (retired CU) |
|  |  | 732038 - Water taxi or crew support |
|  |  | 761050 - Chartered Boat Tours |
| Longshore | 4883 - Support activities for water transportation | 732020 - General wharf operations |
|  | 4885 - Freight transportation arrangement | 732023 - Loading or unloading of goods (not elsewhere specified) |
|  |  | 732025 - Bulk terminal |
|  |  | 732026 - Marine container terminal |
|  |  | 732036 - Stevedoring |
|  |  | 732040 - Harbour commission, port authority, or marine piloting |

**Note:** Please note that these are not one-to-one in some instances. In some instances, a NAICS industry code may map to multiple classification units and the same can be said for some classification units.
