# Supplementary material for: Injury and Return to Work Among Maritime Workers in British Columbia, Canada
Source: New Solut. 2025 Mar 4;35(1):47–59. doi: 10.1177/10482911251316325 (PMC11954171; doi:10.1177/10482911251316325)
Supplement: sj-docx-2-new-10.1177_10482911251316325 - Supplemental material for Injury and Return to Work Among Maritime Workers in British Columbia, Canada [file sj-docx-2-new-10.1177_10482911251316325.docx]

**Supplementary Table 2.** Online Survey Exclusion Criteria.

| **Phase I: 1216 surveys** |  |  |
| --- | --- | --- |
| **Exclusion criteria (any of the below factors)** | **Reason** | **n** |
| Consent | Ethics requirement | 105 |
| Agree to submit (no or blank) | Ethics requirement | 16 |
| Eligibility: Not a maritime worker in the seafaring, longshoring, fishing or aquaculture sectors in British Columbia at some point in the last ten years | Eligibility | 126 |
| Eligibility: Did not experience at least one work-related injury or illness (physical or mental) while working as a maritime worker in British Columbia | Eligibility | 69 |
| Eligibility (based on young or impossible ages) | Eligibility | 3 |
| Impossible cases* |  | 344 |
| Duplicate text^+^ |  | 121 |
| **Total removed** |  | **715** |
| **Phase II: 501 surveys** |  |  |
| **Exclusion criteria** | **Reason** | **n** |
| Recaptcha Score less than 0.5 |  | 30 |
| Start Time identical |  | 365 |
| Unusual character or nonsensical text |  | 11 |
| Duration* |  | 19 |
| Insufficient completion^+^ |  | 18 |
| Text within one field duplicated across >1 survey |  | 94 |
| Highly similar surveys |  | 9 |
| Activity at the time of injury appears nonsensical |  | 12 |
| Experiences to share appear nonsensical |  | 2 |
| Suggested improvements to RTW processes appear nonsensical |  | 5 |
| Dubious ethnicity (landed immigrant AND First Nations or Status Indian) |  | 74 |
| **Total removed (any of the two factors)** |  | **218** |
| **Phase III: 283 surveys** |  |  |
| **Exclusion criteria** | **Reason** | **n** |
| Start times identical AND Stop times within 45 seconds |  | 88 |
| Improbable injury characteristics |  | 2 |
| BC residents who applied for compensation from another province |  | 4 |
| Reported no time off from work BUT did not return to work after the injury |  | 2 |
| **Total removed** |  | **94** |
| **Surveys included for analysis** |  | **187** |

**Phase I**: *Impossible cases: Age at injury did not match year of injury
 ^+^Duplicate text: Two or more surveys with identical text entries in at least two text boxes

**Phase II**: *Completed in less than 206 seconds (lower 2.5 percentile calculated from the 501 surveys that passed the first screening)
 ^+^Less than 30% of survey completed

**Note**: Prior to analysis, 1291 completed surveys were evaluated for inclusion by one data manager. Validated data cleaning strategies (exclusion criteria) were used to identify possible fraudulent surveys completed by survey bots or bad actors. A three-phase hierarchical screening strategy was applied . During the first phase of screening, surveys were excluded from analysis if the participant failed to consent or agree to submit the survey or did not fulfill the eligibility criteria clearly indicated in the consent form. Additionally, impossible cases were excluded where reported age at injury or onset of symptoms did not match the calculated age at injury (reported current age - calculated number of years since injury). Finally, surveys were excluded if two or more text fields were identical to those of at least one other survey. Phase II of screening excluded surveys that contained at least two of Phase II criteria listed in Supplementary Table 2. Phase III of screening excluded surveys with identical start times and stop times that were within one minute of one another. Any surveys that reported bone fractures or amputations injuries associated with only “minor” or “major” symptoms were removed, as were surveys with dubious reported ethnicity claims.

**Supplementary Table 3.** Top 10 Occupations by Sector Among Time-loss Claims Used in the Work Disability Analysis.

|  | N | % |
| --- | --- | --- |
| **Fishing** |  |  |
| 8262 – Fishermen/women | 533 | 50.7 |
| 8441 – Fishing vessel deckhands | 289 | 27.5 |
| 7532 – Water transport deck and engine room crew | 62 | 5.9 |
| 7384 – Other trades and related occupations, not elsewhere classified | 38 | 3.6 |
| 9463 – Fish and seafood plant workers | 29 | 2.8 |
| 8261 – Fishing masters and officers | 21 | 2.0 |
| 9618 – Labourers in fish and seafood processing | 20 | 1.9 |
| 6322 – Cooks | 12 | 1.14 |
| 2274 – Engineer officers, water transport | 11 | 1.1 |
| 2148 – Other professional engineers, not elsewhere classified | 5 | 0.5 |
| **Aquaculture** |  |  |
| 8613 - Aquaculture and marine harvest labourers | 206 | 27.4 |
| 2221 - Biological technologists and technicians | 199 | 26.5 |
| 0823 - Managers in aquaculture | 118 | 15.7 |
| 9463 - Fish and seafood plant workers | 50 | 6.7 |
| 9618 - Labourers in fish and seafood processing | 34 | 4.5 |
| 8441 - Fishing vessel deckhands | 32 | 4.3 |
| 7532 - Water transport deck and engine room crew | 18 | 2.4 |
| 7452 - Material handlers | 14 | 1.9 |
| 8262 - Fishermen/women | 11 | 1.5 |
| 6733 - Janitors, caretakers and building superintendents | 7 | 0.9 |
| **Seafaring** |  |  |
| 7532 - Water transport deck and engine room crew | 1,326 | 41.1 |
| 7533 - Boat and cable ferry operators and related occupations | 363 | 11.3 |
| 2273 - Deck officers, water transport | 190 | 5.9 |
| 2274 - Engineer officers, water transport | 172 | 5.3 |
| 6711 - Food counter attendants, kitchen helpers and related support occupations | 137 | 4.3 |
| 6322 – Cooks | 124 | 3.8 |
| 6524 - Ground and water transport ticket agents, cargo service representatives and related clerks | 74 | 2.3 |
| 7312 - Heavy-duty equipment mechanics | 71 | 2.2 |
| 6611 – Cashiers | 65 | 2.0 |
| 8616 - Logging and forestry labourers | 62 | 1.9 |
| **Longshore** |  |  |
| 7451 - Longshore workers | 3,278 | 70.7 |
| 7452 - Material handlers | 420 | 9.1 |
| 7312 - Heavy-duty equipment mechanics | 141 | 3.0 |
| 7511 - Transport truck drivers | 137 | 3.0 |
| 7302 - Contractors and supervisors, heavy equipment operator crews | 116 | 2.5 |
| 7311 - Construction millwrights and industrial mechanics | 94 | 2.0 |
| 8616 - Logging and forestry labourers | 73 | 1.6 |
| 7241 - Electricians (except industrial and power system) | 44 | 1.0 |
| 7521 - Heavy equipment operators (except crane) | 33 | 0.7 |
| 7237 - Welders and related machine operators | 30 | 0.7 |
